# Carrageenan delays cell cycle progression in human cancer cells in vitro demonstrated by FUCCI imaging

**DOI:** 10.1186/s12906-016-1199-5

**Published:** 2016-08-04

**Authors:** Eka Sunarwidhi Prasedya, Masao Miyake, Daisuke Kobayashi, Akihiro Hazama

**Affiliations:** 1 Department of Cellular and Integrative Physiology, Fukushima Medical University, Fukushima, Japan; 2 Faculty of Mathematics and Natural Science, Mataram University, Mataram, Indonesia

**Keywords:** Carrageenan, Algae, Cancer, Cell cycle, Human cervical carcinoma cells, Human umbilical vein endothelial cells, Fluorescence ubiquitination-based cell-cycle indicator

## Abstract

**Background:**

Carrageenan is a sulfated polysaccharide that exists in red seaweeds recently shown to have anticancer properties. Previous findings show various effects of carrageenan suppressing tumor cell growth. One of the hallmarks of cancer is uncontrolled proliferation, a consequence of loss of normal cell-cycle control, that underlies tumor growth. Recently there is an increasing interest in potential anticancer agents that affect cell cycle in cancer cells. Thus, in this study we investigated the effects of carrageenan on the tumor cell cycle.

**Methods:**

Using human cervical carcinoma cells (HeLa) cells as and human umbilical vein endothelial cells (HUVEC), the cytotoxic effects of kappa carrageenan (k-CO) and lambda carrageenan (λ-CO) at the concentrations of 250–2500 μg/mL were observed. Cell viability was determined using the MTT assay while cell death rates were determined using staining with calcein-AM/propidium iodide. Cell-cycle profile and progression were demonstrated with HeLa cells expressing FUCCI (fluorescence ubiquitination-based cell-cycle indicator) probes (HeLa-FUCCI).

**Results:**

Carrageenan had no significant effect on HUVEC (normal cells). In contrast both forms of carrageenan were cytotoxic towards HeLa cells (cancer cells). Furthermore, according to cell-cycle analysis with FUCCI cells, the cell cycle of HeLa cells was delayed in specific phases due to different carrageenan treatments.

**Conclusion:**

Considering these results, it could be suggested that carrageenan affects the cell-cycle of HeLa cells not only by arresting the cell cycle in specific phases but also by delaying the time needed for the cell to progress through the cell cycle. Additionally, different types of carrageenans have different effects on cell cycle progression. This effect of carrageenan towards cancer cells could possibly be developed into a tumor cell-specific anticancer agent.

## Background

Cancer is the leading cause of death worldwide, accounting for more 8.2 million deaths in recent years [1]. Many polysaccharides have been isolated from mushrooms, fungi, yeast, algae, lichens and plants in the search for potential anticancer drugs. The biological activities of these polysaccharides have attracted considerable attention in the biotechnology and medical fields [2]. In the search for novel compounds with antitumor properties, marine bioresources have become particular interest given their unique bioactivities [3]. Marine algal cell walls were reported to contain sulfated polysaccharides, which are not found in land plants and may have specific functions in ionic regulation [4]. Later studies revealed sulfated polysaccharides from marine algae have many biological and physiological activities including anticoagulant [5], antithrombotic [6], anti-inflammatory [7], antiviral [8], and activities [9].

A sulfated polysaccharide from algae that has been recently studied because of its interesting bioactivities is carrageenan. Carrageenan is a highly sulfated polysaccharide found in marine red algae of the family Rhodophyceae [10]. Carrageenan is used as a stabilizer, gelling agent, thickener, binder and additive in various food and pharmaceutical industries. Carrageenans are composed of linear chains of D-galactopyranosyl units linked via alternated (1 → 3)-β-D-and (1 → 4)-α-D-glucoside [11], in which sugar units have one or two sulfate groups. From the commercial point of view, the most important carageenans can be categorized into kappa (k-), iota (i-), and lambda (λ-) carrageenans, which differ in the number and position of the sulfate groups. Analysis of their structures can be performed by acidic hydrolysis, for which methods have been developed based on reductive hydrolysis [12–14]. Additionally recent studiesshowthatcarrageenan, exhibits many biological and physiological activities besides its antitumor potential [15], including anticoagulant [16, 17], antithrombotic [18, 19], and anti-inflammatory properties [20, 21]. However, in the present study we would like to demonstrate carrageenan mechanism in affecting tumor cell cycle. Previous findings found that carrageenan has the potential to arrest the cell cycle in certain stages such as G2 [22] or S phase [23]. Many conventional anticancer treatments kill cells regardless of whether these cells are normal or cancerous. Based on the discovery that cell cycle characteristics of cancer cells are different compared to normal cells, potential antitumor agents that are able to affect the cell cycle could be a good target for antitumor drug research. Thus, we suggest it would be important to study how carrageenan affects the cell cycle of human cancer cells. In the present study we demonstrate for the first time cell cycle progression of effected human cancer cells by kappa carrageenan (k-CO) and lambda carrageenan (λ-CO) with fluorescence ubiquitination-based cell cycle indicator (FUCCI) that was developed by Sakaue-Sawano et al. [24]. With FUCCI, we are able to observe cell-cycle progression in real time by measuring the expression of an orange-red fluorescent protein by G0/G1 cells and a yellow-green fluorescent protein by S/G2/M cells.

## Method

### Cell culture and treatment

The cell line that was used in this experiment for cytotoxicity analysis was Human Carcinoma Cervical Cell lines (HeLa) for human tumor cell models and Human Umbilical Vein Endothelial Cells (HUVEC) for human normal cell models. Cell cycle progression was observed by HeLa cells (HeLa-FUCCI) expressing the FUCCI (fluorescent ubiquitiniation-based cell cycle indicator) probes provided by the RIKEN BioResource Centre (RIKEN BRC). HeLa cells were maintained in Dulbecco’s Modified Eagle’s Medium from Sigma-Aldrich supplemented with 10 % fetal bovine serum (FBS), at 37 °C in 5 % CO_2_ humidified atmosphere. HUVEC were cultured in Molecular, Cellular, and Developmental Biology (MCDB) 131 medium supplemented with 10 ng/mL epidermal growth factor, 10 μg/mL heparin and 10 % FBS.

### Carrageenan treatment

HeLa and HeLa-FUCCI cells were seeded at a density of 2x10^5^ cells/well in 96 well plates containing Dulbecco’s Modified Eagle’s Medium supplemented with 10 % FBS. HUVEC were cultured in MCDB 131 medium supplemented with 10 ng/mL epidermal growth factor, 10 μg/mL heparin and 10 % FBS. After overnight incubation, k-CO and λ-CO (purchased from Sigma-Aldrich) were added to each assay at concentrations of 250,500,1000, or 2500 μg/mL were added to each assay and maintained at 37 °C in a water saturated atmosphere containing 5 % CO_2_. Cells were incubated in the presence of k-CO and λ-CO for 72 h before cell count analysis.

### Cell growth inhibtion assay

Cell growth conditions of HeLa and HUVEC as a result of exposure to k-COand λ-CO were determined using the Methyl Thiazolyl Tetrazolium (MTT) assay in 96 well plates after carrageenan treatment for 3 days. Cell counting was performed Flexstation 3 Multi_mode Microplate Reader at an absorbance of 450 nm. The viability of the cells was calculated according to the following equation:

$$ \mathrm{Cell}\ \mathrm{viability}\ \left(\%\right) = \left(\left(\mathrm{Abs}460\ \mathrm{sample}\ \hbox{--}\ \mathrm{Abs}460\ \mathrm{blank}\right)/\left(\mathrm{Abs}460\ \mathrm{untreated}\ \hbox{--}\ \mathrm{Abs}460\ \mathrm{blank}\right)\ \mathrm{x}\ 100\ \%\right) $$

Manual cell count were also conducted using a hemocytometer and triplicate readings were recorded. Inhibitory activity expressed as the 50 % inhibition concentration (IC_50_) is the concentration of carrageenan that can inhibit cell growth by 50 % [25]. The IC_50_ values were calculated using the following formula to determine the regression equation y = a + bx.

### Quantification of live and dead cells

To quantify the ratio of live/dead cells, cells were stained with the fluorescent probes calcein AM and propidium iodide (PI). Because of the permeable ability of the cell membrane, calcein AM was used to stain viable cells, while PI was used to label dead cells HeLa and HUVEC were plated onto 35 mm^2^ glass-bottom culture dishes and cultured overnight to achieve cell adhesion. Then cells were treated with different concentrations (250,500,1000, or 2500 μg/mL) of K-CO and λ-CO. After 72 h treatment, the cells were washed three times with a PBS, followed by incubation with a PBS solution mixed with 2 μL calcein AM at 37 °C for 15 min. Finally, cells were stained with 2 μL PI before visualization under. BZ-9000 fluorescence microscope (Keyence, Osaka, Japan). The dead cell ratio was calculated using Image J software [26].

### Cell cycle profile analysis

Cell cycle profiles of the cells following treatment with k-CO and λ-CO were observed using HeLa-FUCCI cells over72h. FUCCI was developed to visualize the dynamics of cell cycle progression. HeLa-FUCCI cells express two fusion proteins : monomeric Kusabira Orange 2 (mKO2) fused to amino acids 30–120 of Cdt1, and monomeric Azami Green fused to amino acids 1–110 of Geminin. This combination of fused proteins causes cells to emit red fluorescence at the early G1 phase immediately after mitosis, and emit green fluorescence in S/G2/M phases. After 72 h treatment of carrageenan on HeLa-FUCCI cells cultured in 35 mm^2^ glass bottom dishes, scanning microscopy was performed with the Bz-9000 fluorescent microscope (Keyence). Scanning and image acquisition were controlled using Bz-II analyzer software (Keyence). The tracing data were imported to Image J for quantification of cells emitting fluorescence of each cell cycle phase.

### Time-lapse Imaging

For a more accurate observation of cell cycle progression due to carrageenan treatment, we set up time-lapse imaging observation with 10 min interval for 72 h. HeLa-FUCCI cell cycle progress was captured using a BIOREVO BZ-9000 fluorescence microscope (Keyence). During imaging, cells were cultured in 35 mm^2^ glass-bottom dishes, held in an incubation chamber to maintain temperature at 37 °C with humidified atmosphere containing 95 % air/5 % CO_2_. Average time of cells from six independent observations to complete cell-cycle phases were calculated for cell-cycle time estimation.

## Results

### Effects of k- and λ- carrageenan on cell growth of normal human cells in vitro

**Fig. 1:**
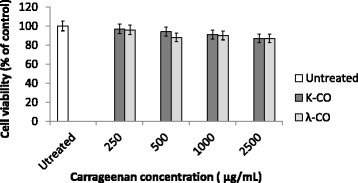
MTT-assay of HUVEC cells treated with K-CO and λ-CO. HUVEC cells were treated with K-CO and λ-CO in 72 h. Cell viability was determined by MTT assay. Results are means ± SD, n = 3 independent observations

The aim for the discovery of potential anticancer agents is that there must be a difference in the killing rate between normal cells and tumor cells. Thus, we sought to determine if carrageenan had cytotoxic effects towards normal human cells, using HUVEC. According to MTT assay readings, both carrageenans had no significant cytotoxic effect towards HUVEC suggesting little effect on the viability of l normal human cells viability (Fig. 1).

### Effects of K- and λ- carrageenan on cell growth of human cancer cells cells in vitro

**Fig. 2:**
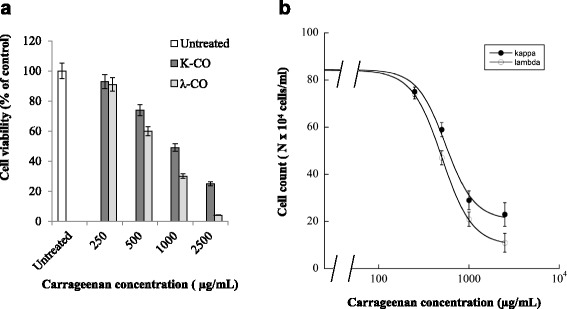
Cytotoxic assay of HeLa cells treated with K-CO and λ-CO. **a**. HeLa cells were treated with K-CO and λ-CO in 72 h. Cell viability was determined by MTT assay. Results are means ± SD, n = 3 independent observations. **b**. To confirm the result of previous MTT-assay, total cell number of carrageenan treated HeLa cells were determined with Haemocytometer cell counter. Exposure of k-CO and λ-CO was associated with inhibited cell growth in exposed vs untreated cells and with exposure to higher carrageenan concentration. Results are means ± SD, n= 3 independent observations

Both types of carrageenan were seen to affect human cancer cell growth significantly. Figure 2a displays MTT assay results illustrating decreased cell viability of HeLa cells exposed with k-CO and λ-CO over a 72 h period. Furthermore, cell growth rates were determined by total cell count of the cells using a haemocytometer. Both k-CO and λ-CO suppressed HeLa cell growth as cell number decreased as carrageenan concentration increased (Fig. 2b). Both k-CO and λ-CO demonstrated IC_50_ values of 550.8 ± μg/mL and 475 ± 12 μg/mL, respectively (Table 1). From these results, it could be assumed that λ-CO possesses stronger cytotoxicity properties compared with k-CO.

**Table 1:** Inhibition carrageenan concentrations causing 50 % cell death (IC_50_) of 72 htreatment

Treatment	IC_50_ (μg/mL)
k-CO	550.8 ± 7.6
λ- CO	475 ± 12

### Viability staining assay

Viability staining was necessary for further confirmation of the current result. Viability staining is an essential tool to obtain a clear picture of cell viability towards treatment of potential drugs. The cytotoxic properties of k-CO and λ-CO were investigated further using a double-labeling procedure with the fluorescence markers calcein-AM and PI, which simultaneously stain live and dead cells in green and red, respectively. Intracellular esterases in live cells can convert the virtually nonfluorescentcell-permeable calcein-AM into the intensely fluorescent calcein. PI enters cells with damaged membranes and enhances fluorescence by binding to DNA, thereby producing a bright red fluorescence in dead cells [27]. HeLa cells were treated with k- and λ- carrageenan at concentrations of 250–2500 μg/mL for 72 h then loaded with calcein-AM and PI before observation with fluorescence microscopy. The population of live (calcein-positive) and dead (PI-positive) cells can be easily differentiated.

**Fig. 3:**
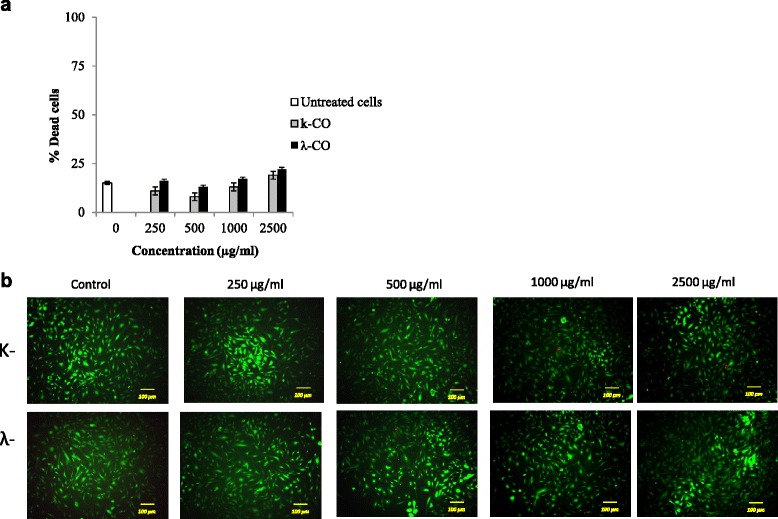
Viability staining of HUVEC cells treated with k-CO and λ-CO. **a**. Dead cell ratio of HUVEC cells exposed with k-CO and λ-CO previously stained by calcein-AM/PI, quantified using imageJ software. Results are means ± SD, n= 3 independent observations. **b**. Viability staining of HUVEC cells treated with concentrations of k-CO and λ-CO 250 μg/ml to 2500 μg/ml showed that λ-CO induces anti-proliferative effects resulting in lower cell density. PI staining (red) indicates dead cells and Calcein AM staining (green) indicates viable cells (scale bar, 100 μm)

**Fig. 4:**
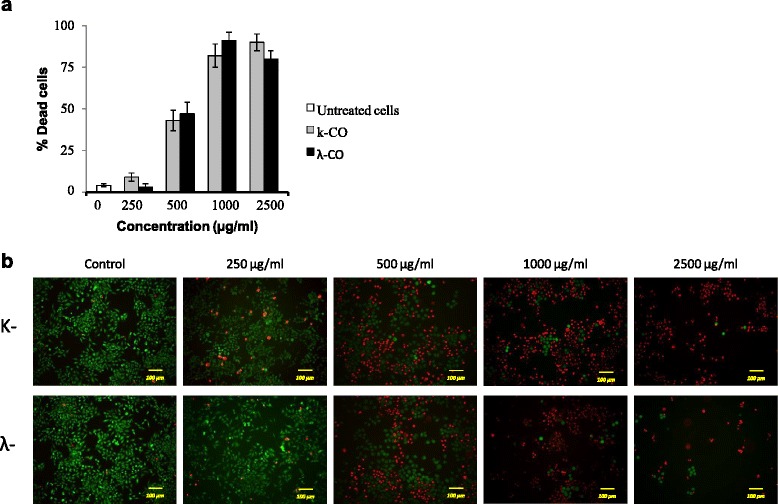
Viability staining of HeLa cells treated with k-CO and λ-CO. **a**. Dead cell ratio of HeLa cells exposed with k-CO and λ-CO previously stained by calcein-AM/PI, quantified using imageJ software. Results are means ± SD, n= 3 independent observations. **b**. Viability staining of HeLa cells treated with concentrations of k-CO and λ-CO 250 μg/ml to 2500 μg/ml showed that λ-CO induces anti-proliferative effects resulting in lower cell density. PI staining (red) indicates dead cells and Calcein AM staining (green) indicates viable cells (scale bar, 100 μm)

As shown in Fig. 3a, there was no increase in cell death ratio of HUVEC treated with carrageenan compared with untreated cells. Viability staining images of HUVEC cells with Calcein-AM/PI also resulted in no significant difference between cells treated with carrageenan and untreated cells (Fig. 3b). Conversely, an increased cell death ratio was observed in HeLa cells treated with both carrageenan treatments (Fig. 4a). Treatment with k-CO resulted in a higher percentage of cell death in a dose-dependent fashion. At maximum concentrations dead cell percentage of k-CO treated cells were higher compared with λ-CO. In contrast, we assumed λ-CO possessed stronger toxic properties towards HeLa cells. Thus, we estimated λ-CO treatment with λ-CO would result in a higher percentage of dead cells. However, this disparity was explained by images from fluorescence microscope showing live images of carrageenan treated cells stained with calcein-AM/PI (Fig. 4b). Observed cells treated with λ-CO were seen to result have a lower cell confluence in a concentration dependent manner. Considerable differences in the cell densities of cells treated with both carrageenans were noticed at maximum concentration. Cells treated with λ-CO for 72 h struggled to reach cell confluence compared to with k-CO, showing a strong inhibition towards HeLa cell proliferation.

### Cell-cycle effects

**Fig. 5:**
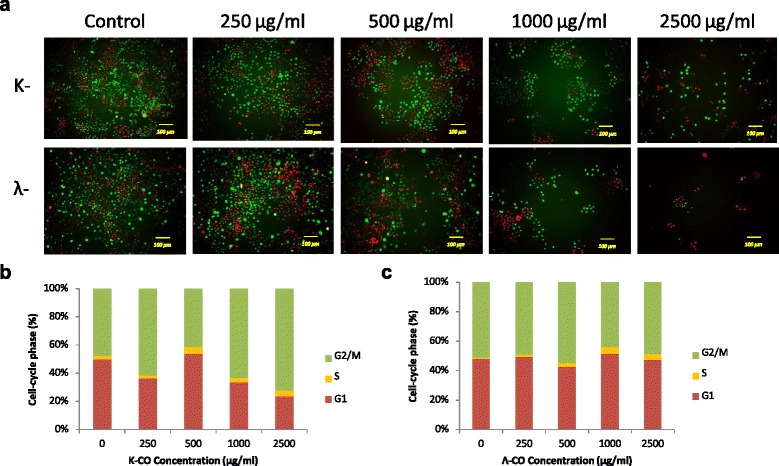
k-CO and λ-CO effects on cell cycle. **a**. Fluorescent images of FUCCI cells treated with k-CO and λ-CO in 72 h (scale bar, 100 μm). **b**. Percentages of cells expressing specific cell phase based on fluorescence color in 72 h after κ-carrageenan treatment **c**. Percentages of cells expressing specific cell phase based on fluorescence color in 72 h after λ-carrageenan treatment

We observed the cell cycle profiles of HeLa cells expressing FUCCI (HeLa-FUCCI) treated with increasing concentrations of k-CO and λ-CO for 72 h. Untreated control HeLa-FUCCI cells exhibited a combination of orange-red and yellow-green nuclei (Fig. 5a) with high cell density. Increased concentration of both k-CO and λ-CO were seen to result in decrease of HeLa-FUCCI cellular density. Cell cycle profiles of k-CO treated cells showed increased arrest in the G2/M phase as green cells (G2/M phase expressing cells) (Fig. 5b). However λ-CO treatment was seen to have no effect on the cell-cycle of HeLa cells, as there were no significant differences between cell ratios of untreated and treated cells (Fig. 5c). Thus further investigation was needed regard on cell-cycle arrest points in carrageenan treated HeLa cells.

### Live cell imaging of cell cycle progress

**Fig. 6:**
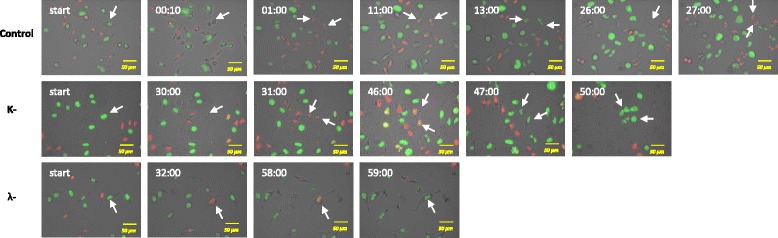
Time lapse imaging of Fucci cells treated with k-CO and λ-CO. Analyse of cell cycle periods via real time lapse imaging of Fucci cells at the transition from G2 as starting phase under carrageenan treatment. Images were taken at a time interval of 10 minutes to observe time length of each cell phase. Displayed times are means from 6 observed cells (scale bar, 50 μm)

To obtain more clear information on the effects of carrageenan towards cell-cycle progress in HeLa cells, we set up time-lapse imaging observation for HeLa-FUCCI cells exposed to k-CO and λ-CO at 1000 μg/mL. Images were taken every 10 min for 72 h and the length of time for each cell phase was measured. Exact lengths of time for each cell phase altered by carrageenan treatment would provide a more effective conclusion regarding cell cycle profiles. As seen in Fig. 6, the lengths of time needed for cells to complete a specific cell-cycle phase were compared. Times displayed are averaged from six independent observations. Untreated cells required approximately 26 ± 0.67 h (Table 2) to complete one cell cycle continuously dividing more than two times. While cells treated with k-CO and λ-CO required a longer time to complete cell cycle. Cells treated with k-CO were seen to require a longer time to complete the G2/M phase, correlating with the previous result which showing a higher ratio of cells in the G2/M phase. Lengths of time of G2/M phase cells treated with k-COwere also seen to show double the time compared with G1 phase time. Cells treated with λ-CO needed a longer time to complete one cycle, approximately 59 ± 4.6 h. This was the result of elongated times for both G1 and G2/M phases, which resulted in a delay in cell-cycle progress overall compared with k-CO treatment which was 50.2 ± 2.9 h. This result explains the previous finding that λ-CO was seen to have no effect on the ratio of cells in cell-cycle phases. Delayed time in both phases (G1 and G2/M) which time lengths were similar resulted in an equal ratio cell in both cell cycle phases. Furthermore, compared with cells treated with k-CO, most cells treated with λ-CO were unable to undergo cell division. Cell cycle progress seems to continue as FUCCI cells change color, except the cells are unable to divide and would later die. However, cells treated with k-CO were able to divide at least once before cell death. Based on these results, we suggest that λ-CO potentially possesses a stronger effect on suppressing tumor cell proliferation and cell division compared with k-CO.

**Table 2:** Time required for cells to undergo phases of cell cycle in treatment of k-CO and λ-CO (1000 μg/mL). Data presented are means of ± SD, *n* = 6 independent observations

Treatment	Time length (h)				
	G0/G1	G1	G1/S	S/G2/M	1 Cycle
Untreated cells	1.5 ± 0.7	9.3 ± 1.8	1.7 ± 0.8	14.1 ± 1.9	26.6 ± 0.6
K-carrageenan	1.0 ± 1.1	16.8 ± 4.0	2.2 ± 1.2	30.1 ± 7.3	50.2 ± 2.9
λ-carrageenan	1.3 ± 0.4	26.5 ± 4.9	2.5 ± 0.2	32.5 ± 5.1	59 ± 4.6

## Discussion

Carrageenan is a polysaccharide that exists in the cell walls of marine red algae and is widely used in studies concerned with its antitumor and cytotoxic activities [10]. Previous findings show carrageenan as a potential antitumor agent [28–30]. Considering one of the hallmarks of cancer is uncontrolled proliferation, a consequence of the loss of normal cell-cycle control, there has been a. increasing interest in potential anticancer agents that affect the cell-cycles of cancer cells [31]. Thus, in this study we investigated how carrageenan affects tumor cell cycle.

In this study we demonstrated cytotoxic effects of carrageenan towards cell cycle of human cancer cells in HeLa expressing FUCCI probes [24]. Two types of carrageenan, kappa (k-CO) and lambda (λ-CO) carrageenan were used because sulfate contents vary in each type of carrageenan [32]. These sulfated moieties in saccharides are believed to play an important role in manifestation of beneficial bioactivity [33]. Thus cytotoxic properties of λ-CO which has more sulfate groups might differ from k-CO. Cytotoxic effects are generally considered as effects of a compound resulting in cell damage or death [34]. Our current results show dissimilar properties of k-COand λ-CO in effecting cell proliferation, cell death ratio, and cell cycle progression.

In vitro cytotoxicity tests are mainly performed to screen potentially toxic compounds that affect basic molecular functions such as cell growth. For acute toxicity measurement, 3 h of exposure is sufficient to reulst in toxic effects in the cell [35]. In the case of measuring cell proliferation, the time of exposure should be 72 h, because that amount of time is required to obtain about three cell divisions [36]. The HeLa cell line chosen as models for this investigation is an immortal cell line generally used in toxicity tests. The ability of this cell line to proliferate rapidly makes it possible to conduct several experiments aimed mainly at detection of biological activity of test substances and also provide long-term culture [37]. The cytotoxic effects of compounds can be classified by the IC_50_ values. IC_50_ values less than 100 μg/mL indicate a potentially cytotoxic compound. IC_50_ values in the range of 100–1000 μg/mL are considered to correspond with moderate cytotoxic effects, and compounds with IC_50_ values greater than 1000 μg/mL are considered non-toxic to the cells [38, 39]. In this study, k-CO and λ-CO both were found to cause moderate toxic effects on HeLa cells because IC_50_ values were in the range of 100–1000 μg/mL (Table 2). However λ-CO potentially possesses stronger cytotoxic effect compared with k-CO based on IC_50_ values. λ-CO was reported to have higher sulfate content than k-CO which results in stronger antioxidant activity. Furthermore, high antioxidant activity may potentially result in anti-proliferative effects [40]. However, further studies are needed to confirm the correlation between sulfate content and anti-proliferative properties. Live:dead cell assays were performed to examine cell death through determination of intracellular esterase activity and plasma membrane integrity. The data showed k-CO resulted in a higher ratio of dead cells after staining with calcein-AM and PI. However, images of HeLa cells treated with λ-CO displayed lower cellular confluence implying cellular proliferation is suppressed by the anti-proliferative potential of λ-CO. The findings of the current study are consistent with those of other studies that showed cell death induced via cellular toxicity caused by carrageenan treatment in human tumor cells [41]. To determine the cytotoxic effects of carrageenan on human normal cells, we used HUVEC. Both k-CO and λ-CO showed no significant cytotoxic effects towards HUVEC viability. We expected cancer cells to be generally resistant to apoptosis compared with normal human cells. However, as seen in responses to potential anticancer agents in cell cultures, most cancer cell lines are more sensitive to apoptosis compared with normal cell lines [42, 43].

Cell cycle arrest and cell death are two important mechanisms involved in anti-cancer drug development. Uncontrolled cellular proliferation is a general characteristic of all cancer cells, and the blockade of the cell cycle is regarded as an effective strategy for eliminating cancer cells. Many chemotherapeutic agents have shown anti-proliferative effects via arresting cell division at certain checkpoints in the cell cycle. The concept of cell cycle-mediated cell death has gained increasing attention, as targeting this pathway may provide an opportunity to overcome drug resistance, decrease mutagenesis and reduce toxicity.

**Fig. 7:**
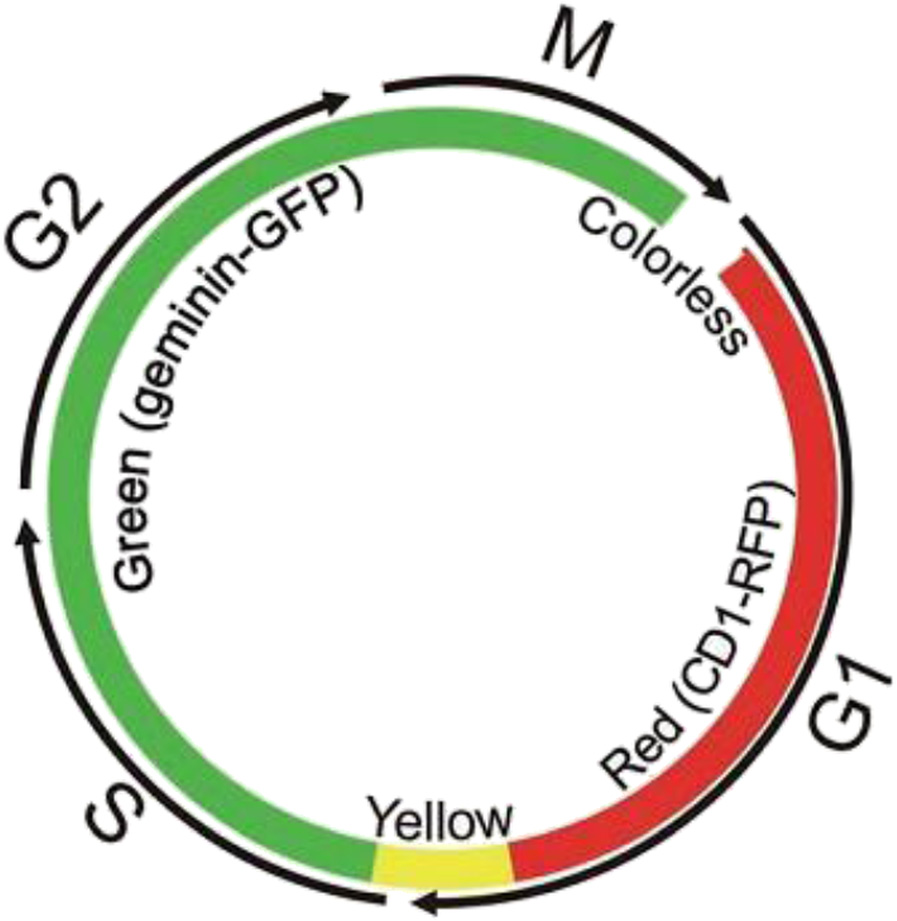
Dynamic color change of HeLa-Fucci cell cycle Sensor. Fucci is a fluorescent, two-color sensor of cell cycle progression and division in live cells. Cells change from red in the G1 to yellow in the G1/S interphase and green in S, G2, and M phases, as geminin and Cdt1, fused to one green and red fluorescent proteins, respectively are expressed at specific points in the cell cycle

In the present study we demonstrated a different method in determining the cell-cycle profile via a modified fluorescent indicator of cell-cycle progression (Fig. 7) expressed in HeLa cells. Observation using fluorescence microscopy revealed clear distinct red, green and a small fraction of doubly fluorescent cells [44]. Our results based on cell cycle ratio showed a diverse distribution of G1 and G2/M phase cells in untreated cells in 72 h. Cells treated with K-CO showed cell-cycle arrest at the G2/M phase. The increase in the ratio of cells in the G2/M phase was linier in relation to the increase in concentrations of K-CO. Interestingly, λ-CO did not show any correlation with cell-cycle arrest as a result of increased concentrations based on the cell phase ratio. Our experiment using live-imaging of FUCCI cell-cycle progression following treatments with carrageenan provided a clearer understanding regarding this uncertainty. Time lapse imaging of individual FUCCI cells treated by carrageenan was conducted to measure precise cell cycle period. Cells treated with k-CO demonstrated a longer time in both the G1 and G2/M phases of the cell cycle compared with untreated cells. However, G2/M phase time was double the time of the G1 phase in cells treated with k-CO. This correlated with the previous result of k-CO treatment stimulating cell-cycle arrest in G2 phase because of a lengthening of the G2/M phase. Treatment with λ-CO showed an even longer period in each cell cycle checkpoints. Elongation of both the G1 and G2/M phase which were almost at similar in length, in cells treated with λ-CO explains the relatively equal ratio of G1 and G2/M cells. Notably, cells treated with k-CO were largely able to continue cell cycle progress to each phase and undergo cell division, only the cell phase periods were longer than control. In contrast, cells treated with λ-CO mostly struggled to continue the cell and hardly underwent division. This finding supports the previous result of suppressed cell growth following treatment with λ-CO. In conclusion both carrageenans induce lengthening of the period in certain phases of the cell cycle, generating a longer time to complete the cycle compared with controls. These results, suggest a different mechanism in both carrageenans affecting tumor cell proliferation.

## Conclusion

Our study findings suggest that carrageenan significantly inhibits HeLa growth not only by arresting the cell cycle in specific phases but also by causing a delay in cell cycle progress. Furthermore, both λ-CO and k-CO have different effects on the cell cycle in HeLa cells. k-CO was seen to delay the cell cycle in G2/M phase while λ-CO stalled the cell cycle in both G1 and G2/M phase, resulting in a longer cell cycle compared with either k-CO treated and untreated cells. Furthermore, λ-CO displayed suppression for the ability of the cell to divide, demonstrating a strong anti-proliferative effect. In contrast, carrageenan shows no significant effects towards HUVEC. However, in vivo studies and trials on other human cancer and normal cell lines are needed to confirm these results for more advanced development of carrageenan as a potential anticancer agent.

## Abbreviations

%, percentage; μg/mL, microgram/milliliter; Abs, Absorbance; Calcein-AM/PI, calcein-acetoxymethyl diacetylester/propidium iodide; DMEM, Dulbecco’s Modified Eagle’s Medium; EGF, epidermal growth factor; FBS, fetal bovine serum; FUCCI, fluorescence ubiquitination-based cell cycle indicator; h, hours; HeLa, human carcinoma cervical cell lines; HUVEC, human umbilical vein endothelial cells; IC_50_, inhibitory concentration of 50 %; mAG, monomeric azami green; MCDB, molecular, cellular, and developmental biology; mKO2; mm^2^, square millimeter; monomeric kusabira orange 2; MTT, methyl thiazolyl tetrazolium; ng/mL, nanogram/milliliter; nm, nanometer; °C, degree celcius; RIKEN BRC, RIKEN BioResource Centre; k-CO, kappa carrageenan; λ-CO, lambda carrageenan
